# Correction: Luo et al. Dietary Anti-Aging Polyphenols and Potential Mechanisms. *Antioxidants* 2021, *10*, 283

**DOI:** 10.3390/antiox15050556

**Published:** 2026-04-28

**Authors:** Jing Luo, Hongwei Si, Zhenquan Jia, Dongmin Liu

**Affiliations:** 1 Guangdong Provincial Key Laboratory of Food, Nutrition and Health, Department of Nutrition, School of Public Health, Sun Yat-sen University, Guangzhou 510080, China; luojing27@mail.sysu.edu.cn; 2 Department of Human Sciences, Tennessee State University, Nashville, TN 37209, USA; hsi@tnstate.edu; 3 Department of Biology, University of North Carolina, Greensboro, NC 27402, USA; z_jia@uncg.edu; 4 Department of Human Nutrition, Foods and Exercise, College of Agricultural and Life Sciences, Virginia Tech, Blacksburg, VA 24061, USA

There was an error in the published paper [1] regarding the inclusion of Ref. [30] and the associated statement in the main text. Subsequent evaluation has determined that the referenced work is unreliable and should not be cited.

Accordingly, Ref. [30] has been removed from the reference list, and the corresponding sentence in the main text has been deleted.

The authors apologize for any inconvenience caused.

The correction has been approved by the Academic Editor. These changes do not affect the conclusions of the paper.
